# Outcomes following CyberKnife robotic radiosurgery for pituitary adenomas—a large single-centre study

**DOI:** 10.3332/ecancer.2024.1803

**Published:** 2024-11-28

**Authors:** Kamran Saeed, Kaynat Siddiqui, Hafiza Fatima Aziz, Fatima Shaukat, Shazia Kadri, Aneeta Ghulam Muhammad, Aneela Darbar, Tariq Mahmood

**Affiliations:** 1 Cyberknife and Tomotherapy Center, Jinnah Postgraduate Medical Center (JPMC), Karachi 75510, Pakistan; 2 Dr. Ziauddin Hospital, Karachi 74700, Pakistan; 3 The Aga Khan University Hospital, Karachi 74800, Pakistan

**Keywords:** CyberKnife, radiosurgery, pituitary adenomas, acromegaly, Cushing’s disease, nonfunctional adenomas

## Abstract

**Introduction:**

The role of stereotactic radiosurgery (SRS) in pituitary adenomas (PAs) is evolving especially considering its safety. Existing literature is hampered by limited sample sizes and short-term follow-ups, impeding its preeminence in the clinical and radiological outcomes. We propose a comprehensive, single-centred study to evaluate the outcomes following CyberKnife stereotactic radiosurgery (CK SRS) for PAs in a larger patient population, incorporating meticulous clinical and radiological follow-up.

**Methods:**

This is a retrospective cohort study of 278 cases of PAs that underwent CK SRS from 2013 to 2021. Based on their endocrinology profile, they were classified as functional adenomas (FA) and non-functional adenomas (NFA). We assessed pre and post-CK SRS clinical, visual, hormonal and radiological parameters and the associated toxicity. Where applicable, data were compared using the Independent t-test, chi-square test, Fisher Exact and Mann-Whitney *U* test. A *p*-value <0.05 was considered significant.

**Results:**

The median age of the patients was 40.13 ± 12.61 years (111 female and 167 male patients). The median prescribed radiosurgery dose was 25.0 ± 5.0 Gy into 3 or 5 fractions. The median follow-up time was 12 months (IQR 20). Data were grouped into NFA (169, 60.8%) and FA (109, 39.2%). After adjusting for patients lost to follow-up, post-CK SRS visual perimetry improved in 80.4% of patients and tumour size reduced in 78.6% of the study population. Seventeen patients with NFA and nine with FA manifested new-onset hormonal deficiencies. No statistically significant differences were seen in post-CK SRS visual outcomes and hormone deficiency groups.

**Conclusion:**

CK SRS is effective and safe for managing PAs, achieving tumour control and preserving visual function with minimal toxicity. Extended follow-up is needed to evaluate post-SRS toxicity and hypopituitarism.

## Introduction

Pituitary adenomas (PAs) constitute a heterogeneous group of intracranial tumours, representing approximately 14%–22% of primary benign brain tumours [1]. Their classification as functional or non-functional is contingent upon their hormonal secretions. Incidental discovery or clinical attention is prompted by symptoms arising from mass effects, such as headaches, visual field defects, apoplexy or endocrinopathies [2].

Surgery is the gold standard modality for non-functional and functional adenomas (FA) except in cases of prolactinomas that require mainly medical management and often yield optimal curative outcomes. The selection of an optimal treatment modality is contingent upon factors such as the tumour’s anatomical location, its proximity to optic pathways and the patient’s clinical status. However, stereotactic radiosurgery (SRS) has demonstrated promise in managing residual and recurrent adenomas, particularly in cases where surgery is declined or tumours are deemed inoperable [3, 4]. Among SRS modalities, CyberKnife stereotactic radiosurgery (CK SRS) stands out as an advanced technology that uses robotics to deliver radio surgical doses in a frameless manner, eliminating the need for skull fixation and minimal patient discomfort, distinguishing it from gamma knife procedures [5].

While prior studies have indicated favourable outcomes for CK SRS in both FA and non-functional adenomas (NFA) on tumour reduction or stability, vision preservation and neuroendocrine control [6–8] – existing literature is hampered by limited sample sizes and short-term follow-ups, impeding the definitive establishment of CK SRS preeminence in the clinical and radiological outcomes of PAs. Consequently, we propose a comprehensive, single-centred study to evaluate the outcomes following CK SRS for PAs in a larger patient population, incorporating meticulous clinical and radiological follow-up.

## Methods

### Patient information

The data were retrospectively gathered from the clinical records of 278 patients from 2013 to 2021 at the CyberKnife Robotic Radiosurgery Centre in Karachi, Pakistan. All data were collected following Institutional Review Board guidelines. All patients received CK SRS as an adjunct for residual or recurrent tumours or upfront in cases where surgery was not considered as a first-line treatment modality due to co-morbidities, critical location or clinical status of the patient.

### Radiosurgery technique and parameters

Patients underwent treatment on the CyberKnife G4 machine model equipped with independent rapid integrated shaping. It utilises a 6- mega-voltage lightweight linear accelerator mounted on a fully articulated robotic arm. Treatment planning involved using Multiplan software (Accuray precision 3.3) for inverse planning. Patients were positioned supine and immobilised using a thermoplastic face mask. Treatment plans were generated at a workstation using image data from contrast-enhanced computed tomography (CT) scans with a slice thickness of 1 mm, supplemented with gadolinium-enhanced magnetic resonance imaging (MRI). The gross tumour volume (GTV) and critical structures were delineated in each consecutive CT slice. No additional margin was applied to the GTV to derive the clinical target volume and planning target volume.

### Clinical assessment

The initial assessment incorporated formal visual field testing (perimetry), conducted by an ophthalmologist, gadolinium-enhanced MRI scans and hormonal assessment by an endocrinologist. In our clinic, all patients had follow-ups, which included MRI with contrast and perimetry. For non-functional pituitary adenomas, follow-ups were conducted at 3 or 6-month intervals during the first 2 years, followed by annual evaluations thereafter. In contrast, patients with functional pituitary adenomas (FPA) were monitored every 8 months until hormonal remission was achieved, after which they transitioned to annual follow-ups. We evaluated all post-CK SRS variables at the latest follow-up. The departmental radiologist evaluated the MRI scans. Any reduction in tumour size on MRI was considered tumour reduction, while the absence of any visible contrast-enhancing lesion on MRI was taken as complete tumour resolution. Tumour stabilisation was defined as no change in tumour size compared to pre-CK SRS scans. Similarly, vision changes detected through perimetry were classified as improved, stable or deteriorated accordingly.

### Hormonal assessment

The post-treatment endocrine follow-up was determined by the patient’s endocrinologist, considering the pre-treatment endocrine status and the adenoma’s functional status. We evaluated the latest hormonal profile. In our study, we defined biochemical remission in FPA as the normalisation of hormone levels. Partial reduction was defined as a decrease in specific hormone levels in FPA, although not within their normal ranges. Additionally, an increase in hormone levels was defined as levels exceeding their pre-CK SRS values. All patients underwent an evaluation for post-treatment hypopituitarism.

### Statistical analysis

Data were analysed by using SPSS version 26. For quantitative variables mean ± SD or median (IQR) were reported based on normality. However, qualitative variables were reported as frequency and percentages. Statistical comparisons were done using parametric or non-parametric tests, as appropriate. Continuous variables were analysed using the independent *T*-test or Mann–Whitney *U* test depending on the normality of the distribution. Normality was examined by the use of the Shapiro–Wilk test. Categorical variables were analysed by Pearson’s chi-squared test or Fisher’s exact test, as appropriate. For the comparison of post-CK perimetry and post-CK radiology with continuous variables, ANOVA or Kruskal–Wallis tests were applied, as appropriate.

## Results

At the time of treatment, the median age of the patients was 40.13 ± 12.61 years, ranging from 8 to 79 years. The cohort comprised 111 female and 167 male patients. Eighty-four percent (*n* = 234) of the study population had previous surgery. The median follow-up time was 12 (IQR 20) months. Detailed patient demographic data is presented in Table 1.

**Figure 1.**
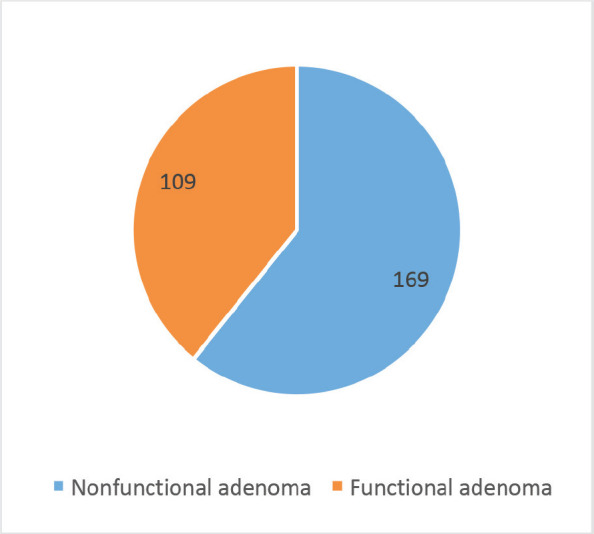
Types of PAs.

**Figure 2.**
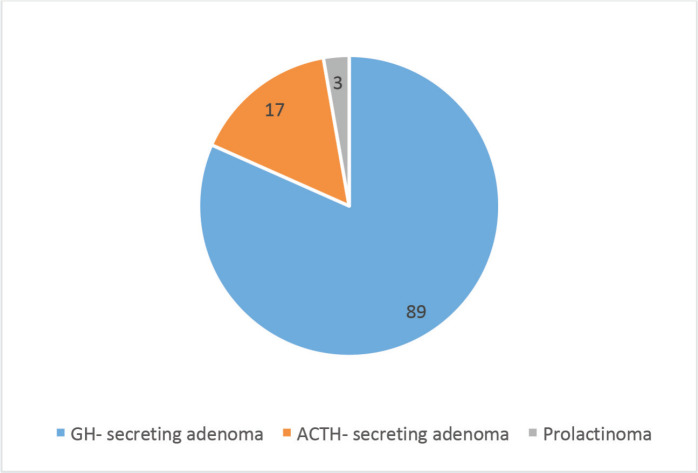
Sub-groups of FA.

The study included 169 (60.8%) NFA and 109 (39.2%) FA (Figure 1). The most common FA was growth hormone (GH)-secreting (*n* = 89, 81.7%) followed by Adrenocorticotrophic hormone (ACTH)-secreting adenomas (*n* = 17, 15.6%) and medically refractory prolactinomas (*n* = 3, 2.8%) (Figure 2). The median prescribed dose was 25.0 ± 5.0 Gy into 3 or 5 fractions. The median GTV was 7,143.5 (IQR 5937.6) mm.

### Visual outcomes

Among a cohort of 278 patients, the pre-CK SRS perimetry was available for 265 patients. Out of these, partial visual impairment was present in 239 (86%) cases. However, only 189 (67.9%) patients provided post-CK SRS perimetry for comparison. Among these, the visual field improved in 152 (80.4%), while remained stable in 35 (18.5%) patients. Two (1.1%) instances of deteriorated vision were recorded post-intervention, one of which was attributed to pituitary apoplexy. Although post-CK SRS perimetry improved in patients with larger GTV (*p*- value = 0.18), we found no statistically significant correlation between post-CK SRS perimetry and tumour type, history of previous surgery, gender, age and radiosurgery parameters (Table 2).

### Radiological outcomes

**Figure 3.**
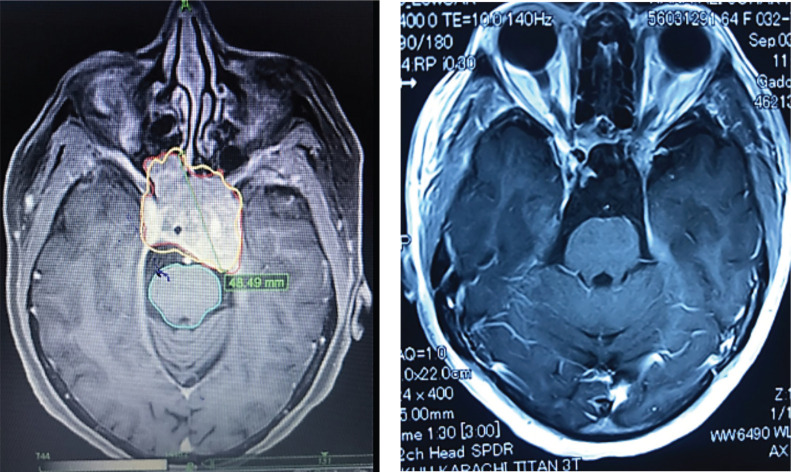
(a): A case of large pituitary macroadenoma before receiving CyberKnife radiosurgery. (b): Near complete resolution of pituitary macroadenoma of the same patient after receiving CyberKnife radiosurgery in the follow-up period.

During the follow-up period, only 257 participants provided MRI scans, representing 92.4% of the cohort. Among these individuals, 202 (78.6%) had a reduction in tumour volume, 13 (5.1%) had complete resolution of enhancing lesion and 42 (16.3%) had no interval change in tumour size throughout the follow-up period (Figure 3a and b). We observed that post-CK SRS tumour reduction was more in the larger GTV group (*p*-value = 0.068). Furthermore, there was no statistically significant correlation between post-CK SRS radiological outcomes and tumour type or radiosurgery parameters (Table 3).

### Hormonal outcomes

Among the cohort of 278 patients, 87 individuals (32%) exhibited pre-CK SRS hormonal deficiencies, primarily resulting from the tumour or post-surgical resection. Notably, following the treatment, 224 (80.2%) patients followed with the endocrinologist and, therefore, were assessed for any new onset hypopituitarism. We found that 17 (10.1%) cases of NFA and 9 (8.3%) cases of FA manifested new-onset hormonal deficiencies and 199 (71.58%) patients had normal endocrine profiles in the post-CK SRS follow-up. Moreover, there is no statistically significant correlation with age or previous surgery (Table 4).

The endocrinological monitoring of acromegaly patients involved the assessment of insulin like growth factor -1 and GH levels. Among the 77 cases included in the follow-up analysis, biochemical remission was observed in 28 cases (36.4%), while 43 cases (55.8%) showed a partial reduction in hormone levels. Conversely, hormone level elevation was documented in 6 patients (7.8%). Similarly, 14 individuals with Cushing’s disease underwent follow-up evaluations based on serum cortisol levels. Among these cases, seven (50%) achieved biochemical remission, while six (42.9%) exhibited a partial reduction in hormone levels. However, in one instance (7.1%), there was an observed increase in hormone levels during the follow-up period. Furthermore, two (66.7%) cases of medically refractory prolactinoma demonstrated a partial reduction in hormone levels followed by a singular case of biochemical remission following CK SRS (Table 5).

### Post SRS toxicity

Among the 278 cases, post-SRS complications were encountered in 6 patients (2.6%). These complications encompassed mild sensory disturbances in the facial region or occurrences of sharp intermittent pain at the lateral facial region that was managed conservatively. Importantly, no instances of extraocular motor deficits were observed in any patient during the follow-up period. Five patients experienced mortality during the follow-up period. One patient succumbed to end-stage renal disease, while another developed uncontrolled diabetes leading to fatal complications. Both of these patients had co-morbid conditions prior to CK SRS. Additionally, a patient with Cushing’s disease died due to uncontrolled ACTH levels as he did not seek further treatment. Two patients experienced cerebrovascular accidents, leading to subsequent mortality.

## Discussion

The management of FA requires a multidisciplinary approach. In the case of prolactinomas, medical interventions serve as the primary treatment modality, with further intervention considered only when the condition becomes medically refractory. Conversely, for acromegaly and Cushing’s disease, surgery is considered the gold standard of treatment. After initial therapeutic measures, residual or recurrent disease is effectively managed through SRS. In instances where surgical intervention is contraindicated due to coexisting medical conditions, patient refusal or heightened surgical risks attributed to critical tumour locations, radio-surgical intervention is employed as a primary treatment modality for these tumours [4].

We observed that a significant number of cases in our study had improved perimetry in the follow-up period which may be due to the hypofractionated schedule of the CK- SRS as highlighted by Meral *et al* [9]. In their study of 31 patients with acromegaly treated with hypofractionated CK SRS, they observed 100% tumour control along with 86.7% hormonal remission. Furthermore, three patients had improved optic neuropathy. However, 32.3% of patients developed hypopituitarism in the follow-up period [9]. In another study by Abdali *et al* [10] 41 post-surgical residual ACTH-secreting adenomas, were treated with single fraction SRS. They reported that at a median follow-up of 48.8 months, none of their patients had any visual deterioration and 60.97% of cases achieved biochemical remission at 14 months [10]. Similarly, a cohort of 53 patients with perioptic PAs within 3 mm of optic apparatus, were treated with fractionated CK SRS where none experienced visual decline [4]. The authors also reported a case of pituitary apoplexy, which was observed in our study as well. Sala *et al* [7] shared their experience with CK SRS for invasive GH-secreting macroadenomas, where they observed no visual deterioration during the follow-up period of 43.2 months. Furthermore, Kajiwara *et al* [11] studied 21 patients with PAs who underwent CK SRS with a mean dose of 14.3 ± 4.5 Gy at a mean follow–up of 35.3 ± 10.7 months. They reported worsened visual acuity in one patient 2 years post-CK SRS due to cystic expansion of the tumour [11]. However, a study of 20 consecutive patients, treated with hypofractionated CK SRS, was followed for 26.6 ±10.5 months and did not report any visual deterioration post SRS [12]. In a retrospective study of ACTH-secreting adenomas, 7 cases were observed for different outcome measures where none reported worsening of vision in the follow-up period [13]. In a large study of perioptic tumours treated with hypofractionated CK SRS, it was observed that among 40 patients with PAs, one patient experienced visual deterioration in the absence of any tumour progression at 17 months follow-up that later resolved with the treatment in the next few months [14]. In a study of 50 patients undergoing CK SRS, 8% (*n* = 4) had post-SRS change in visual function [15]. In an extensive literature review by Kajiwara *et al* [16] there were no visual deficits reported in any study of PAs treated with CK SRS [16]. This has been further supported by Iwata *et al* [17] who studied the long-term effects of hypofractionated CK SRS in 52 patients with GH-secreting adenoma. He concluded that by using hypofractionation and low toxicity dose for the optic apparatus, one can avoid any worsened visual acuity in the extended post-SRS period [17]. Similarly, the visual outcome of 21 patients with residual or recurrent PAs reported no deterioration in vision post-CK SRS delivered in a multi-session fashion [6]. Our study is per the existing literature where only 1.1% of cases experienced a deteriorated vision in the post-CK SRS period; however, 80.4% of cases had visual improvement, whereas 18.5% of patients remained stable.

In one of the recent studies of GH-secreting PAs by Romero-Gameros *et al* [18] biochemical remission was achieved in 12.2% post-CK SRS, while 33.3% of the patients required additional medical treatment. Notably, 54.4% of the cohort exhibited persistent biochemical activity [18]. Fourteen (24.56%) patients developed panhypopituitarism at the end of the follow-up; however, there were no cases of optic pathway toxicity or any cerebrovascular accident [18]. Apaydin *et al* [19] determined that within a cohort of 38 cases undergoing either GK SRS or CK SRS, the median duration for the biochemical control rate was 52.6% whereas, the time to remission was 15 months. In a study by Ehret *et al* [15] a total of nine patients (18%) achieved biochemical remission, 24 patients (48%) maintained biochemical control and 17 patients (34%) exhibited uncontrolled biochemical profiles at the last follow-up. Moreover, three patients (6%) manifested new-onset hypopituitarism [15].

Similarly, within a retrospective analysis encompassing 53 patients, Plitt *et al* [4] determined that the hormonal status remained unchanged in 98.1% of cases following CK SRS. Notably, a singular patient exhibited a deteriorated hormonal profile attributed to pituitary apoplexy at 4 months post-SRS. Furthermore, there was a 75% rate of hormonal control for FA [4]. In a small study of 22 GH- secreting adenomas, 18.1% exhibited biochemical control, whereas 40.9% of patients achieved complete control. However, 6 new patients developed new onset pituitary deficiency at 31.6 ± 14.5 months [7]. Puataweepong *et al* [14, 20] investigated a cohort of 40 patients diagnosed with perioptic PAs who underwent hypofractionated CK SRS. Their findings, based on a prescribed dose of 25 Gy administered in 5 fractions over a 38.5-month follow-up period, revealed hormonal control in 54% of patients, with no occurrences of new-onset hypopituitarism. However, one patient with a prolactinoma experienced an increase in tumour size during the post-SRS follow-up period [20]. All the existing studies conclude hypopituitarism is the late toxicity of CK SRS as observed in 26 cases in our study cohort.

## Conclusion

In summary, the results of our study support the efficacy and safety of CK SRS in managing PAs, emphasizing its role in achieving tumour control and preserving visual function. The utilisation of SRS is particularly beneficial for protecting critical optic pathways and providing maximum tumour control with a minimal toxicity profile. However, there is still a need for extended follow-up to evaluate further for post-SRS toxicity and hypopituitarism. We anticipate that our outcomes will contribute significantly to paving further way for establishing the standard treatment protocol for PAs.

## Limitations

There are a few limitations to our study. This is a retrospective study and several cases were lost to follow-up in various instances. The follow-up period should be extended to evaluate further long-term complications.

## Conflicts of interest

The authors declare that they have no conflicts of interest.

## Funding

The author(s) received no financial support for the research, authorship or publication of this article.

## Figures and Tables

**Table 1.** Baseline and treatment characteristics of patients undergoing fractionated CyberKnife radiosurgery for PAs.

Characteristics	*n* (%)
Total cases	278
Sex	
Male	167 (60.1)
Female	111 (39.9)
Mean age (years)	40.13 ±12.61
Median follow-up in months (IQR)	12(20)
Previous surgery	234 (84.8)
Deaths in the follow-up period among the total cohort	5(1.8%)
Type of adenoma	
Non-functional	169 (60.8)
Functional	109 (39.2)
GH-secreting	89 (32)
ACTH-secreting	17 (6.1)
Medically refractory prolactinoma	03 (1.1)
Pre-CK SRS visual deficit	
Partially affected	239 (90.2)
No vision	05 (1.9)
Normal vision	21 (7.9)
Missing data from the total cohort	13(4.7%)
Pre CK SRS hypopituitarism	87 (32.0)
New onset hypopituitarism	
NFA	17 (12.3)
FA	09 (10.3)
Post-CK SRS toxicity	6 (2.6)
Radiosurgery prescribed dose (Gy)	
Mean	25.0
IQR	5.0
Fractions	5 or 3
GTV (mm)	
Median	7,143.5
IQR	5,937
CI	
Median	1.31
IQR	0.13
nCI	
Median	1.35
IQR	0.14
HI	
Median	1.25
IQR	0.04
Coverage	
Median	97.02
IQR	3.62

IQR: Inter-quartile range; GH: Growth hormone; ACTH: Adrenocorticotrophic hormone; CK SRS: CyberKnife stereotactic radiosurgery; NFA: Nonfunctional adenoma; FA: Functional adenoma; Gy: Gray; GTV: Gross tumour volume; CI: Conformity index; nCI: New conformity index; HI: Homogeneity index

**Table 2.** Correlation of post radiosurgery visual outcomes with other variables.

Post CK SRS visual outcomes (*n* = 189)**				
Variable	Improved (*n* = 152)	Deteriorated (*n* = 2)	Stable (*n* = 35)	*p*-value
Age (mean ± SD)	40.09 ± 12.48	47.5 ± 4.95	40.26 ± 13.72	0.714^¥^
Gender***				
Male	91 (79.82)	0 (0)	23 (20.18)	0.176^ƿ^
Female	61 (81.33)	2 (2.67)	12 (16)	
Tumour size***				
Macro	142 (79.33)	2 (1.12)	35 (19.55)	0.596^ƿ^
Micro	4 (100)	0 (0)	0 (0)	
Tumour type				
Functioning	51 (78.46)	0 (0)	14 (21.54)	0.453^ƿ^
Non-functioning	101 (81.45)	2 (1.61)	21 (16.94)	
Pre CK SRS GTV	7,360.84 (5,127.63–1,1195.27)	3,995.65 (3,995.65–3,995.65)	6,340.28 (4,384.96–1,1142.56)	0.18^£^
Radiation Parameters				
CI (mean ± SD)	1.33 ± 0.13	1.25^α^.	1.32 ± 0.1	0.762^¥^
nCI (mean ± SD)	1.38 ± 0.12	1.32^α^.	1.36 ± 0.1	0.773^¥^
HI (median with IQR)	1.25 (1.23–1.27)	1.2 (1.2–1.2)	1.25 (1.23–1.28)	0.322^£^
C (mean ± SD)	96.48 ±2.91	94.83^α^	96.68 ± 2.31	0.794^¥^

^¥^ANOVA; ^£^Kruskall Wallis; ^ƿ^Chi-Square test, ^α^ SD was not calculated due to low sample

**89 patients did not follow-up with perimetry; ***Missing cases did not provide post-treatment perimetry A *p*-value of <0.05 was considered significant

CK SRS: CyberKnife stereotactic radiosurgery; SD: Standard deviation; GTV: Gross tumour volume; CI: Conformity index; nCI: New conformity index; HI: Homogeneity index; C: Coverage

**Table 3.** Factors associated with change in tumour volume (post CK SRS radiology).

Post CK SRS tumour volume (*n* = 257)**				
Variable	Reduced (*n* = 202)	Resolve (*n* = 13)	Stable (*n* = 42)	*p*-value
Tumour size				
Micro	189 (78.42)	12 (4.98)	40 (16.6)	0.325^ƿ^
Macro	7 (87.5)	1 (12.5)	0 (0)	
Tumour type				
Non functioning	74 (77.89)	7 (7.37)	14 (14.74)	0.399^ƿ^
Functioning	128 (79.01)	6 (3.7)	28 (17.28)	
Pre CK GTV^#^	7,529.12 (5,069.64–11,142.56)	5,763.48 (3,144.22–9638.75)	5,782.94 (4,326.18–7,360.84)	0.068^£^
Radiation parameter				
CI^#^	1.31 (1.25–1.38)	1.32 (1.25–1.4)	1.29 (1.25–1.34)	0.475^£^
nCI^#^	1.36 (1.29–1.43)	1.42 (1.29–1.43)	1.33 (1.26–1.4)	0.484^£^
HI^#^	1.25 (1.23–1.28)	1.23 (1.22–1.28)	1.25 (1.23–1.27)	0.553^£^
C^#^	97 (95.23–98.8)	97.25 (96.2–97.89)	96.4 (94.83–99.19)	0.932^£^

^#^Median (IQR); ^£^Kruskall Wallis; ^ƿ^Chi-Square test

**21 cases did not provide post SRS MRI

*p*-value of <0.05 was considered significan

CK SRS: CyberKnife stereotactic radiosurgery; GTV: Gross tumour volume; CI: Conformity index; nCI: New conformity index; HI: Homogeneity index; C: Coverage

**Table 4.** Factors associated with new onset hormone deficiency in the follow-up period.

Variable	*n* = 225**		*p*-value
	No (*n* = 199)	Yes (*n* = 26)	
Age*	39.78 ± 12.56	38.54 ± 13.56	0.660^β^
Gender			
Male	130 (90.28)	14 (9.72)	0.251^ƿ^
Female	69 (85.19)	12 (14.81)	
Previous surgery			
No	28 (93.33)	2 (6.67)	0.364^α^
Yes	170 (87.63)	24 (12.37)	
Type of surgery			
TS+CONVENTIONAL RADIATION	1 (100)	0 (0)	0.197^ƿ^
TS-TF	12 (100)	0 (0)	
TS	146 (87.95)	20 (12.05)	
TF	11 (73.33)	4 (26.67)	
Tumour size			
Macro	186 (87.74)	26 (12.26)	0.292^α^
Micro	8 (100)	0 (0)	
Tumour type			
Functioning	78 (89.66)	9 (10.34)	0.652^ƿ^
Non-functioning	121 (87.68)	17 (12.32)	
Pre CK SRS GTV^#^	7,088.63 (4,750.18–11,142.56)	8,070.82 (4,460.33–13,344.8)	0.419^µ^
Radiation parameter			
CI^#^	1.32 (1.25–1.38)	1.28 (1.25–1.3)	0.050^µ^
nCI^#^	1.36 (1.3–1.43)	1.32 (1.28–1.36)	0.055^µ^
HI^*^	1.26 ± 0.05	1.25 ± 0.03	0.332^β^
C*	96.82 ± 2.34	96.48 ± 2.56	0.527^β^

*Mean ± SD; ^#^Meadian (IQR); ^β^Independent *t*-test; ^ƿ^Chi-square test; ^α^Fischer exact test; ^µ^Mann Whitney *U* test

**53 patients lost to endocrine follow-up

*p*-value of <0.05 was considered significant

TS: Transphenoidal surgery; TF: Transfrontal surgery; CK SRS: CyberKnife stereotactic radiosurgery; GTV: Gross tumour volume;

CI: Conformity index; nCI: New conformity index; HI: Homogeneity index; C: Coverage

**Table 5.** Hormonal outcomes of FPA.

	GH-secreting adenoma *n* (%)	ACTH-secreting adenoma *n* (%)
Patients with follow-up	77*	14**
Partial reduction	43 (55.8)	6 (42.9)
Biochemical remission	28 (36.4)	7 (50)
Increase in hormone	6 (7.8)	1 (7.1)
Mean time for biochemical remission (months)	22.7	32.5

*12 cases lost to endocrine follow-up

**3 cases lost to endocrine follow-up
